# Sperm HSP70: may not be an age-dependent gene but is associated with field fertility in Bali bulls (*Bos sondaicus*)

**DOI:** 10.1590/1984-3143-AR2023-0048

**Published:** 2024-05-03

**Authors:** Dian Tria Fatmila, Berlin Pandapotan Pardede, Tulus Maulana, Syahruddin Said, Yudi Yudi, Bambang Purwantara

**Affiliations:** 1 Study Program of Animal Science, Faculty of Agriculture, Universitas Sumatera Utara, Medan, Indonesia; 2 Research Center for Applied Zoology, National Research and Innovation Agency, Bogor, Indonesia; 3 Division of Reproduction and Obstetrics, School of Veterinary Medicine and Biomedical Sciences, IPB University, Bogor, Indonesia

**Keywords:** age, Bali bulls, fertility, gene, HSP70, semen quality

## Abstract

This study aimed to analyze the characteristics of the HSP70 gene and protein in spermatozoa of Bali bulls of different age groups and to examine its potential as a biomarker determining bull fertility. This study used frozen semen produced from six Bali bulls divided into two groups based on age (≤ 9 years and ≥ 12 years). Parameters of frozen semen quality analyzed included sperm motility and kinetics using computer-assisted semen analysis, sperm morphological defects using Diff-Quick staining, acrosome integrity using FITC-PNA staining, and DNA fragmentation using acridine orange staining. HSP70 gene expression characterization was analyzed using qRT-PCR, and HSP70 protein abundance was analyzed using enzyme immunoassays. Fertility field data were obtained by analyzing the percentage conception rate for each bull based on the artificial insemination service data contained in the Indonesian-integrated system of the National Animal Health Information System (iSIKHNAS). The results showed significant differences (P<0.05) in total and progressive motility, morphological defects of the neck and midpiece, and tail of sperm, and acrosome integrity between the age groups of Bali bulls. HSP70 gene expression and protein abundance showed no significant differences (P>0.05) in different age groups. HSP70 gene expression correlated with fertility rate (P<0.05). Age affected several semen quality parameters but did not affect HSP70 gene expression and protein abundance. The HSP70 gene molecule could be a biomarker that determines the fertility of Bali bulls.

## Introduction

Bali cattle (*Bos sondaicus*) are a native breed of Indonesian beef cattle, domesticated from wild banteng (*Bos javanicus*) in Bali and Java for hundreds of years. Bali cattle have various advantages, such as adaptability to different environmental conditions, unfavorable feed, and a reasonably high carcass percentage, reaching around 51% (Saputra et al., 2017). Besides having good productivity, Bali cattle are also reported to have good reproductive performance and fertility (Purwantara et al., 2012). The various advantages of Bali cattle have made this local cattle breed one of the superior beef cattle alternatives to meet the increasing demand for beef in Indonesia, so an increase in the population and genetic quality of these local cattle is urgently needed. Artificial Insemination (AI) is an assisted reproductive technology that can improve livestock's genetic quality and productivity. AI generally uses frozen semen produced through cryopreservation and from superior bulls, including Bali bulls. Frozen semen used for AI is categorized as of good quality if it has progressive motility of more than 40% (Pardede et al., 2024), the acrosome is intact, not-fragmented DNA, and it contains sperm with abnormal morphology <15% so that they can produce optimal fertility levels (Rezaei et al., 2020). Pardede et al. (2020a) reported that semen quality is closely related to bull fertility. Furthermore, it was reported that apart from semen quality, increasing age can affect semen quality and have an impact on decreasing the fertility of bulls (Pardede et al., 2020b).

Increasing age is one of the factors that can affect the metabolic system in the body that cannot be avoided. Aging can cause a decrease in semen quality (Tsai et al., 2013) and impact decreasing fertility (Kidd et al., 2001). Various species have reported that age affects fertility (Pardede et al., 2020b; Gangwar et al., 2021). Aging can cause a physiological decline in the reproductive organs that can cause disturbances in spermatogenesis. Gonadotropin levels increase, and testosterone levels decrease with aging, decreasing the quantity of Leydig, Sertoli, and germ cells. It reduces reproductive performance, including semen quality and fertility of bulls (Pino et al., 2020). In addition, aging also makes cells vulnerable to oxidative stress (ROS) due to mitochondrial dysfunction (Singh et al., 2019). Reactive oxygen species (ROS), one of the excess oxidant products, can initiate cell damage, leading to apoptosis (Singh et al., 2019) and cause a decrease in semen quality, one of the essential factors that can affect bull fertility.

Although it is one of the essential factors that can affect the fertility of bulls, recent studies have shown that good semen quality does not guarantee optimal fertility in the field. Pardede et al. (2022) reported that although the quality of semen met specific requirements and was considered to be of good quality, three out of six bulls in the field showed a low fertility rate (<50%). Recent research has found various gene and protein molecules in spermatozoa that play an essential role in the reproductive process of bulls, mainly the physiological functions of sperm oocytes, which are believed to be able to predict bull fertility accurately (Özbek et al., 2021; Li et al., 2016; Pardede et al., 2024; Rosyada et al., 2023a). Furthermore, this is due to the involvement of more complex molecular mechanisms in spermatozoa, including the loss of spermatozoa surface protein required for fertilization (Li et al., 2016).

Heat shock protein 70 (HSP70) is a molecule in sperm with a molecular weight of 70 kDa, which has a role in maintaining homeostasis from changes in temperature and oxidative stress (Rosenzweig et al., 2019; Cheng et al., 2016). Agarwal et al. (2020) reported that HSP70 in bull spermatozoa is associated with motility, capacitation, and spermatozoa-oocyte interactions. The abundance of HSP70 protein in several bulls, such as Holstein Friesian bulls (Somashekar et al., 2017), Tharparkar (Rajoriya et al., 2014), and Simmental (Cheng et al., 2016) was found in spermatozoa. It was characterized through a proteomic technology approach. Furthermore, Bashiri et al. (2021) stated that the expression of the HSP gene in spermatozoa contributes to the early development of the embryo, and changes in its expression pattern can cause pregnancy failure and reduce fertility. This study aims to analyze the characteristics of the HSP70 gene and protein in spermatozoa of Bali bulls in different age groups and to examine its potential as a biomarker determining bull fertility, especially in Bali bulls, which has never been reported before.

## Methods

### Ethical approval

The sample used is a commercial product and is not directly related to the bulls used in the study. Every procedure, from storing fresh semen to turning it into frozen semen, is carried out according to the protocol set at the Bali AI center and has complied with every animal welfare principle.

### Experimental animals

The sample used in this study was frozen semen produced at the Regional Artificial Insemination Center, Bali Province, Indonesia. This study only used frozen semen, a commercial product from the AI center ready to be distributed in the field, as the primary sample and did not directly involve bulls. Furthermore, the entire semen collection process was carried out using an artificial vagina for the same period (without seasonal differences), frozen using the same extender. To eliminate any potential for variation in the samples, the bulls were kept in the same environment regarding feeding and handling management. Frozen semen produced from six Bali bulls divided into two groups based on age (≤ 9 years and ≥ 12 years) was used in this study for further analysis. This grouping is based on Zainudin et al. (2014), which state that productive bulls are less than ten years old. At the age of more than ten years, there is a decrease in reproductive ability physiologically and hormonally.

### Frozen semen quality

Frozen sperm from each bull was defrosted in a 37°C water bath for 30 seconds before being placed in a microtube for further analysis. The parameters of semen quality tested in this study were sperm motility and kinetics, morphology, acrosome integrity, and DNA fragmentation. The stages of this study used five biological repeats for each of the tested semen quality parameters. Sperm motility and kinetic analysis were carried out using computer-assisted semen analysis (CASA) with the Sperm-Vision Program (Minitüb, Tiefenbach, Germany), which was linked to Carl Zeiss Microimaging GmbH (Gottingen, Germany) and equipped with a warm stage at 38°C. The parameters analyzed were total motility (TM), progressive motility (PM), curvilinear velocity (VCL), straight-line velocity (VSL), average path velocity (VAP), linearity (LIN), and straightness (STR) (Fischer et al., 2014).

Sperm morphological defects were analyzed using the commercial Diff-Quick Staining Kit (Aurora Scientific, Indonesia), which refers to the method of Tuset et al. (2008). Sperm morphological defects are classified into three groups: head defects, neck and mid-piece defects, and tail defects (Chenoweth, 2005). Analysis of the integrity of the sperm acrosome in this study was carried out using the FITC-PNA staining method, according to Cocchia et al. (2011). The DNA damage of spermatozoa was examined using acridine orange (AO) staining, as described by Pardede et al. (2021).

### HSP70 gene expression analysis

Semen samples thawed in a water bath at 37°C for 30 seconds were then placed in a microtube. Then, RNA was isolated and extracted from each semen sample using the DirectZol RNA kit (Zymo Research, USA), and reverse transcription and cDNA synthesis was performed using the SensiFAS™ cDNA Synthesis kit (Bioline Ltd, UK). Then, the master mix consisting of the SensiFAST™ SYBR® No-ROX kit (Bioline Ltd, UK), forward and reverse primers, nuclease-free water, and cDNA was added to the PCR plate. The primer sequences used for HSP70 gene expression analysis (NM_174344.1) were forward 5'-TTGGGGACAAGTCAGAGAATG-3' and reverse 5'-ATCGTGGGGTTCCTTTTGATG-3'. The HSP70 primer was obtained from literature studies where this primer has been applied and used on local bull breeds, such as Qinchuan (*Bos taurus*) (Zhang et al., 2015a), Madura (*Bos javanicus* x *Bos indicus*) (Rosyada et al., 2023b), and Bali (*Bos sondaicus*) (Pardede et al., 2023) bulls. PPIA (NM_178320.2) was used as an endogenous control, with the primary sequence being forward 5'-ATGCTGGCCCCAACACAA-3' and reverse 5'-CCCTCTCACCTTGCCAAA-3'. Gene expression analysis was performed using the quantitative reverse transcription PCR (qRT-PCR) method, using the Bio-Rad CFX OPUS 96 Real-Time PCR System. This quantification analysis obtained the cycle threshold (C_T_) value representing the nucleic acid copy number. The relative quantification value of HSP70 gene expression was calculated using the 2^-ΔΔ^C_t_ formula, which compared the C_T_ value of the target gene with PPIA.

### HSP70 protein abundance analysis

The frozen sperm sample was thawed in a 37°C water bath for 30 seconds before being centrifuged at 1000 x g for 20 minutes. HSP70 protein abundance was analyzed using enzyme immunoassays (EIA) according to the protocol instructions for use on the bovine HSP70 kit (Cat No. MBS7606199, MyBioSource.com). The absorbance value will be determined using an EIA reader at 450 nm. The abundance of HSP70 protein in a sample can be determined by comparing its value with the standard curve (Mostek et al. 2017).

### Field fertility analysis

The field fertility assessment is determined by the value of the first service conception rate of each bull based on the success of AI. The data are in the form of an AI application using frozen semen straws from bulls used in the study and the results of pregnancy examinations for each inseminated female cow. More than 1,000 data on the success of AI over the last two years were used in the study to analyze the percentage of first service conception rate in each bull using the Pardede et al. (2020a) technique. The first service conception rate was defined as the number of pregnant cows (80–100 days after the first AI service) to all inseminated cows. However, the AI success data used is secondary data reported by AI officers in the field, which is contained and recorded in the Indonesian-integrated National Animal Health Information System (iSIKHNAS) and does not directly involve female cows. The %fertility rate analysis results for each bull sequentially were 74.60%, 78.09%, 75.13% (≤ 9 years bulls), and 73.43%, 74.67%, and 74.50% (≥ 12 years bulls). The analysis results were then used to predict the %field fertility rate for each bull in the study.

### Statistical analysis

The independent t-test was used to analyze data on differences in semen quality, gene expression and protein of HSP70, and field fertility between age groups. The relationship between gene expression and protein of HSP70 with semen quality and field fertility was analyzed using the Pearson correlation test. The correlation pattern between gene expression and protein abundance of HSP70 with field fertility was determined using the Scatter Plot linearity test. Data was analyzed using SPSS program version 25.0 (IBM, Armonk, NY, USA). Data are presented in the mean ± standard error.

## Results

**Figure 1:**
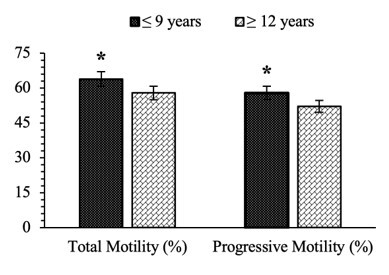
Percentage comparison of spermatozoa's total and progressive motility between the age groups of Bali bulls. The sign (*) for each parameter of semen quality indicates a significant difference (P<0.05) between the age groups of bulls. Data are presented in the mean ± standard error.

The findings revealed a significant variation (P<0.05) in total (63.81±0.84% vs. 57.91±1.50%) and progressive motility (57.89±1.05% vs. 52.14±1.70%) between age groups of Bali bulls (Figure 1). Kinetic parameters of sperm cells, such as VCL, VSL, VAP, STR, and LIN, revealed no significant difference (P>0.05) among Bali bull ages (Table 1).

**Table 1:** Percentage comparison of sperm kinetic parameters between age groups of Bali bulls.

**Age group (years)**	**VCL**	**VSL**	**VAP**	**STR**	**LIN**
	**(μm/s)**	**(μm/s)**	**(μm/s)**	**(%)**	**(%)**
≤ 9	100.50±2.68	51.01±1.43	71.53±1.73	71± 1.00	50.00±1.00
≥ 12	97.77±1.96	49.95±1.68	69.88±1.50	71± 1.00	50.00±1.00

VC: curvilinear velocity; VSL: straight line velocity; VAP: average path velocity; STR: straightness; LIN: linearity.

**Figure 2:**
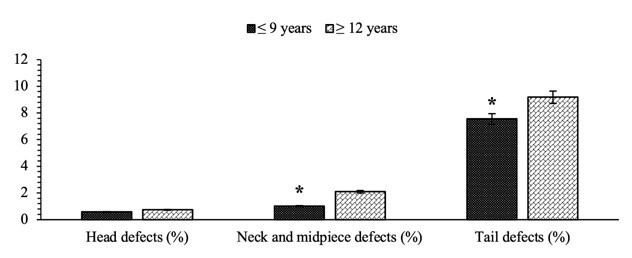
Percentage comparison of sperm morphological defects (head, neck mid-piece, and tail defects) between the age groups of Bali bulls. The sign (*) for each parameter of semen quality indicates a significant difference (P<0.05) between the age groups of bulls. Data are presented in the mean ± standard error.

The results showed that there were significant differences (P<0.05) in the sperm morphological defects, the defects in the neck and midpiece of the sperm (1.01±0.28% vs. 2.10±0.26%) and the tail of the sperm (7.56±0.28% vs. 9.18±0.38%) between the age groups of Bali bulls (Figure 2). At the same time, there was no significant difference (P>0.05) in the head defects (P>0.05) between the age groups (Figure 2).

**Figure 3:**
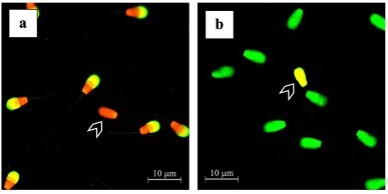
The photomicrograph of sperm in (a) FITC-PNA (acrosome integrity) and (b) AO (DNA fragmentation) staining. Sperm acrosome integrity will be stained with orange fluorescence (arrow). Sperm DNA fragmentation will be stained with yellow-orange fluorescence (arrow).

**Figure 4:**
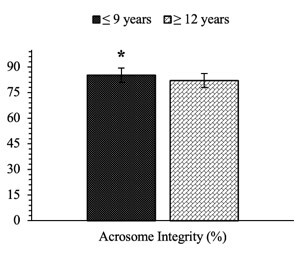
Percentage comparison of acrosome integrity in the age group of Bali bulls. The sign (*) for each parameter of semen quality indicates a significant difference (P<0.05) between the age groups of bulls. Data are presented in the mean ± standard error.

This study found a significant difference (P<0.05) in sperm acrosome integrity analyzed using the FITC-PNA method (Figure 3) between the age groups of Bali bulls (85.14±0.82% vs. 82.01±1.16%) (Figure 4). Sperm DNA fragmentation assessed using the AO staining method (Figure 3) in this study was not significantly different (P>0.05) between age groups of bulls (Table 2). In addition, the percentage of field fertility between the age groups of bulls did not differ significantly (P>0.05) (Table 2).

**Table 2:** Percentage comparison of sperm DNA fragmentation and field fertility between age groups of Bali bulls.

**Age group (years)**	**DNA fragmentation (%)**	**Field fertility (%)**
≤ 9	2.11±0.18	75.94±1.09
≥ 12	2.48±0.21	74.20±0.39

**Figure 5:**
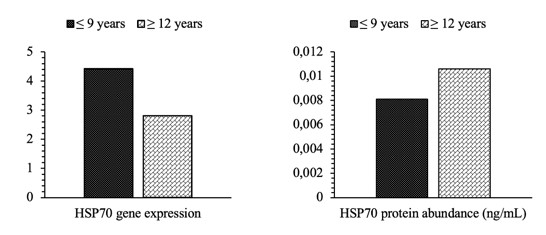
Percentage comparison of HSP70 gene expression and protein abundance in the age group of Bali bulls. Data are presented in mean ± standard error.

**Figure 6:**
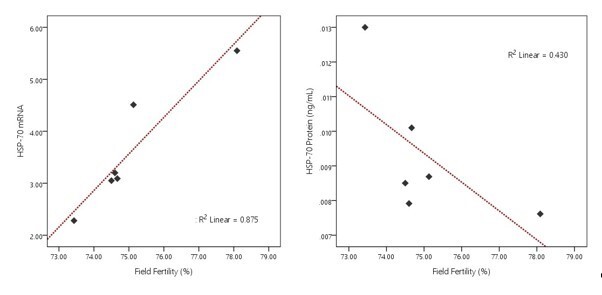
The pattern of linearity relationship between HSP70 gene expression and protein abundance with field fertility.

HSP70 gene expression (4.42±0.62 vs. 2.81±0.26) and its protein abundance (0.0081±0.00021 ng/mL vs. 0.0106±0.00085 ng/mL) in this study showed no significant difference (P>0.05) between the age groups of Bali bulls (Figure 5). The results of the correlation analysis between HSP70 gene expression and semen quality are shown in Table 3. In contrast, the results of the correlation analysis between HSP70 protein abundance and semen quality are shown in Table 4. The results showed that HSP70 gene expression positively correlated with field fertility (P<0.05) (Table 4). The results of the linearity test using the Scatter Plot curve show that only HSP70 gene expression has a linear and positive relationship with field fertility (Figure 6).

**Table 3:** Correlation of HSP70 gene expression to field fertility and semen quality parameters.

**Parameters**	**Correlation coefficient**	**P-value**
HSP70 gene expression vs. field fertility (%)	0.935	<0.006**
HSP70 gene expression vs. TM (%)	0.521	>0.289
HSP70 gene expression vs. PM (%)	0.481	>0.335
HSP70 gene expression vs. VCL (μm/s)	0.283	>0.587
HSP70 gene expression vs. VSL (μm/s)	-0.070	>0.895
HSP70 gene expression vs. VAP (μm/s)	0.102	>0.847
HSP70 gene expression vs. STR (%)	-0.364	>0.478
HSP70 gene expression vs. LIN (%)	-0.338	>0.512
HSP70 gene expression vs. DNA fragmentation (%)	-0.246	>0.638
HSP70 gene expression vs. acrosome integrity (%)	0.753	>0.084
HSP70 gene expression vs. head defects (%)	-0.549	>0.259
HSP70 gene expression vs. neck and midpiece defects (%)	-0.729	>0.100
HSP70 gene expression vs. tail defects (%)	-0.700	>0.122

TM: total motility; PM: progressive motility; VCL: curvilinear velocity; VSL: straight line velocity; VAP: average path velocity; LIN: linearity; STR: straightness. The sign (**) for each parameter of semen quality indicates a significant difference (P<0.01) between the age groups of bulls. Data are presented in the mean ± standard error.

**Table 4:** Correlation of HSP70 protein abundance to field fertility and semen quality parameters.

**Parameters**	**Correlation Coefficient**	**p-value**
HSP70 protein abundance (ng/mL) vs. field fertility (%)	-0.656	>0.157
HSP70 protein abundance (ng/mL) vs. TM (%)	0.065	>0.903
HSP70 protein abundance (ng/mL) vs. PM (%)	0.135	>0.799
HSP70 protein abundance (ng/mL) vs. VCL (μm/s)	0.260	>0.618
HSP70 protein abundance (ng/mL) vs. VSL (μm/s)	0.567	>0.240
HSP70 protein abundance (ng/mL) vs. VAP (μm/s)	0.450	>0.370
HSP70 protein abundance (ng/mL) vs. STR (%)	0.775	>0.070
HSP70 protein abundance (ng/mL) vs. LIN (%)	0.718	>0.108
HSP70 protein abundance (ng/mL) vs. DNA fragmentation (%)	-0.023	>0.966
HSP70 protein abundance (ng/mL) vs. acrosome integrity (%)	-0.602	>0.206
HSP70 protein abundance (ng/mL) vs. head defects (%)	0.425	>0.401
HSP70 protein abundance (ng/mL) vs. neck and midpiece defects (%)	0.258	>0.621
HSP70 protein abundance (ng/mL) vs. tail defects (%)	0.542	>0.266

TM: total motility; PM: progressive motility; VCL: curvilinear velocity; VSL: straight line velocity; VAP: average path velocity; LIN: linearity; STR: straightness.

## Discussion

Fertility is a multifactorial phenomenon influenced by many factors, including the bull factor (Morrell et al., 2017). A decrease in bull fertility can cause substantial economic losses in the livestock sector (Özbek et al., 2021). Selvaraju et al. (2021) stated that several factors, including semen quality, influence bull fertility. In addition to semen quality, bull fertility and reproductive capacity are also affected by age (Narud et al., 2022; Gunes et al., 2016), which can be seen from a decrease in semen quality. As found in this study, there was a decrease in several parameters of semen quality in the group of bulls over 12 years of age. Overall, the motility of spermatozoa in this study was far above the minimum standard (>40%) for frozen semen quality values determined based on the Indonesian National Standard. The field fertility analysis for each Bali bull in this study also showed promising results. Hardjopranjoto (1995) stated that the success of artificial insemination, as measured by the level of fertility in the field, is categorized as good if it reaches a value of 65-75%.

Various studies have reported the effect of age on decreasing semen quality and bull fertility (Carreira et al., 2017). Gunes et al. (2016) reported that aging is correlated with increased oxidative stress, which causes an increase in lipid peroxidation and the formation of ROS in mitochondria. Singh et al. (2019) also stated that oxygen in mitochondria is essential in oxidative phosphorylation, which involves glucose breakdown and ATP formation. ROS plays a critical role in male fertility (du Plessis et al., 2015); low ROS levels are needed for several physiological processes of spermatozoa, but excessive amounts can cause male infertility (Bisht and Dada, 2017). ROS concentrations increase with age, along with a decrease in antioxidant levels (Paul and Robaire, 2013). Excessive ROS production can cause damage to sperm cells (Andrabi, 2009). Furthermore, aging causes histological changes in the testes, such as alteration in the morphology of germ cells, Sertoli cells, peritubular testes, and Leydig cells (Gunes et al., 2016). ROS are widely known in spermatozoa as second messengers in important cellular events related to the fertilization process, such as acrosome reaction, hyperactivation, and spermatozoa-oocyte fusion, so the physiological amount of ROS is essential to maintain cell balance.

Besides impacting increasing oxidative stress, aging is also reported to be associated with decreased tryptophan levels in seminal plasma. Tryptophan is an amino acid that plays a role in 5-hydroxytryptamine synthesis, which supports the acrosome reaction and regulates sperm motility and tyrosine phosphorylation activity (Narud et al., 2022). Chianese and Pierantoni (2021) also reported that aging correlates with poor semen quality, one of which is motility. The restriction of mitochondrial membrane potential, which disrupts the process of forming ATP, which is required for the pattern of movement of spermatozoa, causes a decrease in sperm motility. Previous research has also found that aging reduces sperm motility and kinetic parameters (Hellstrom et al., 2006). It is the same as seen in the study, in which there was a significant decrease (P<0.05) in total and progressive spermatozoa motility in bulls over 12 years of age. Nevertheless, there was no statistically significant difference in the kinetic parameters of spermatozoa between bull age groups.

Sperm morphological defects are influenced by various factors, including environmental and genetic (Chenoweth, 2005; Auger et al., 2016). Saito et al. (2020) reported that aging could cause an increase in sperm morphological defects. The high percentage of sperm morphological defects can cause fertilization failure (Lopes et al., 1998). In this study, sperm neck, mid-piece, and tail defects differed significantly (P<0.05) between the age groups. These two morphological defects of the sperm are parts of the sperm that are related to the function of the movement of the sperm, especially the mid-piece, which is where the mitochondria of the cells are located (Chenoweth, 2005). This finding follows the percentage of total and progressive motility in this research, revealing a drop in quality in the bulls over 12. Chianese and Pierantoni (2021) showed that mitochondria are organelles in cells that are the site for ATP production, which produces the energy needed for sperm to be able to move progressively motile until they reach the site of fertilization.

In contrast, there was no substantial difference in sperm head defects between the age groups (P>0.05). Chenoweth (2005), Carreira et al. (2017), and Kusumawati et al. (2023) reported that sperm head defects, which are significant abnormalities in sperm, are generally caused by various things, such as genetic factors and DNA condensation. High sperm head defects were also reported to correlate with decreased bull fertility (Lemma, 2011). No differences were found in the abnormalities of the sperm heads in this study, possibly due to the condition of the sperm DNA, which was also in good condition. The findings of this study also show that the sperm DNA fragmentation values between ages were not significantly different (P<0.05). In addition, it can be seen that the fertility rate of bulls in this study is still relatively good.

Sperm DNA integrity is essential for the transmission of the genetic code. It is considered a potential marker of male fertility, so high numbers of fragmented spermatozoa will significantly cause fertilization failure and delay embryo development (Mohammed et al., 2015). There wasn't a substantial difference in DNA fragmentation values (P<0.05) between the age groups in this study. It differs from the findings of Carreira et al. (2017) and Gunes et al. (2016), who found a link between age and DNA fragmentation. Pardede et al. (2020b) also reported an effect of age on increasing DNA fragmentation in bulls, although overall, the fragmentation value was still relatively good, which was less than 15%. High sperm DNA fragmentation is often associated with excess ROS production due to cold shock during sperm cryopreservation (Baumber et al., 2003). Oxidative stress, due to an imbalance in ROS production, can cause DNA fragmentation and affect fertility (Razavi et al., 2021). Increased levels of ROS cause harmful effects at the molecular level of sperm because it results in peroxidation of sperm membrane lipids, loss of protein function, and occurrence of DNA fragmentation, which negatively impacts the normal function of sperm (Panner Selvam et al., 2020). In addition, Pardede et al. (2021) also reported an abnormality in the protamine composition in the sperm nucleus, which resulted in high sperm DNA fragmentation. Protamine is the main protein in the core of mature sperm, which wraps and protects the sperm DNA, which, if a disturbance occurs, will have an impact on reducing bull fertility (Pardede et al., 2020c).

Failure of the acrosome reaction process due to damage to the acrosome cap of sperm is one of the fundamental causes that cause infertility in bulls (Rennemeier et al., 2009). The integrity of the sperm membrane and acrosome is critical in fertilization (Yadav et al., 2018). The acrosome is a distinct membranous organelle found in the sperm nucleus's anterior region, which functions in the process of penetration of sperm in the zona pellucida during fertilization (Wang et al., 2014). The proportion of acrosome integrity differs substantially (P<0.05) between ages, according to the findings. It follows what Carreira et al. (2017) reported that aging would cause the acrosome in sperm cells to be more susceptible to various damages. This is because aging is associated with decreased antioxidant protective activity due to the cryopreservation process, which also results in a considerable reduction in glutathione peroxidase and superoxidase dismutase (Carreira et al., 2017). In addition, previous studies also reported another effect of the cryopreservation process, particularly as another impact of cold shock on semen quality, namely a decrease in heat shock protein (Huang et al., 2000; Zhang et al., 2015b; Varghese et al., 2016).

Cold shock is one of the effects of the cryopreservation phase that has been linked. The decrease in heat shock proteins, including HSP70, increased ROS concentrations and oxidative damage to intracellular enzymes (Kurashova et al., 2019). HSP70 is a chaperone molecule usually induced during stress on cells (Reddy et al., 2018). In bovine, the HSP70 protein is widely distributed in spermatogonia, spermatids, and spermatozoa, and its localization can change during spermatogenesis, after ejaculation, as a result of capacitation and acrosome reactions, and during cryopreservation (Kamaruddin et al., 2004). HSP70 plays a role in the activation process of antioxidants, glutathione, taurine, creatine, zinc, vitamin E, vitamin C, and vitamin A and modulates the activation of heat shock factor and the subsequent synthesis of HSP70 (Singh et al., 2019; Kurashova et al., 2019). In addition, the HSP70 protein is considered an essential element for the process of meiosis and maturation of sperm, as well as preventing apoptosis because it can activate the natural immune system and play a role in immunomodulation (Wang et al., 2020). HSP70 also plays a role in sperm-egg membrane interactions (Spinaci et al., 2006). Furthermore, HSP70 can increase membrane fluidity as a sperm protector in the female reproductive tract (Moein-Vaziri et al., 2014).

Overall, the HSP70 gene and protein could be detected in the sperm of the Bali bulls used in this study. Although not significantly different (P<0.05), HSP70 gene expression in bulls aged more than 12 years tended to be lower than in bulls aged less than nine years. It indicates the possibility of a decrease in HSP70 gene expression with increasing age in bulls. Gunes et al. (2016) reported that increasing age would impact oxidative stress, which causes an increase in lipid peroxidation and the formation of ROS in mitochondria. An increase in oxidative stress, which increases ROS, is reported to be associated with a decrease in HSP70 (2019). Although the expression of the HSP70 gene did not vary among ages, the research reveals a strong bond between HSP70 gene expression and field fertility. HSP70 gene expression has a positive correlation (P<0.01) with field fertility, which is also proven by the linearity test using the Scatter Plot curve, where there is a positive and linear relationship between these two parameters (Figure 5). It indicates that it could be a biomarker determining the fertility of bulls. The same finding was also reported by Rajoriya et al. (2014) and Abdulla et al. (2022), who proposed that HSP70 is related to fertility rates. Bashiri et al. (2021) discovered that heat shock protein expression in sperm contributes to embryogenesis and performs a crucial function in fertilization, particularly during sperm-egg membrane interactions. Gene expression patterns or protein content changes can cause conception failure and reduce fertility.

There was no substantial difference (P>0.05) in the abundant HSP70 protein between the ages. In addition, the HSP70 protein does not have a close relationship with the fertility field and shows a negative linear curve (Figure 5). According to Vihinen (2015), initial genetic changes at the DNA and RNA levels can influence the final protein generated, affecting the number of protein molecules. Variations in these proteins could have a wide range of consequences on attributes such as sequence, conformation, stabilization, interaction, activity, concentration, and others (Vihinen 2015). Clancy and Brown (2008) and Indriastuti et al. (2022) also revealed that, in simple terms, protein is the result of translating genetic information from DNA transferred by mRNA through the transcription process. mRNA is subject to a wide range of degradative processes interconnected in translation events to regulate protein expression (Silver and Noble 2012). Not all areas of the mRNA can be translated, particularly the untranslated region close to the 5' end of the molecule (UTR). Differences in the length of the 5' UTR can affect protein binding sites, affecting mRNA stabilization or translation utilization (Clancy and Brown 2008). Translation of mRNA into protein is influenced by various factors, such as post-transcriptional gene silencing (PTGS), mRNA stability, and factors affecting mRNA stability, so a high mRNA level indicates a poor protein level if there are problems in the translation process (Egelkrout et al., 2012).

Correlation test results between the HSP70 gene or protein and semen quality parameters did not show a strong relationship. It is different from previous studies, which found that there is a close relationship between HSP70 and motility (Pardede et al., 2023; Zhang et al., 2015a; Hung et al., 1998), sperm morphological defects (Huang et al., 2000), acrosome integrity (Kamaruddin et al., 2004), and DNA fragmentation (Rajoriya et al., 2014). Varghese et al. (2016) also reported that there was a significant (P<0.05) decrease in HSP70 levels before freezing and after thawing. The difference in HSP70 levels was very substantial from the apical area on the head of cryopreserved sperm and compared to the same site of ​​sperm acrosome before the cryopreservation process. Furthermore, Grzmil et al. (2008) reported that the normal function of spermatozoa from each parameter of spermatozoa quality is managed by much more than one gene or protein and is a complicated task. Disruptions in every gene or protein that regulate the quality attributes of these sperms are very likely to impact decreasing bull fertility, which varies.

## Conclusion

This study found a possible effect of age on a decrease in reproductive capacity, as seen from several parameters of semen quality, which decreased in Bali bulls aged more than 12 years. Bali bull age did not affect HSP70 gene expression and protein abundance. The HSP70 gene molecule has the prospect of being a valuable marker for determining Bali bull fertility.
